# Lack of detection of aluminium‐reactive T‐lymphocytes in patients with SCIT‐induced granulomas

**DOI:** 10.1002/clt2.12378

**Published:** 2024-07-02

**Authors:** Stine Skovbo Hoffmann, Jesper Elberling, Jeanne Duus Johansen, Lars Heede Blom

**Affiliations:** ^1^ Department of Dermatology and Allergy National Allergy Research Centre Herlev and Gentofte Hospital University of Copenhagen Hellerup Denmark; ^2^ Department of Dermatology and Allergy Herlev and Gentofte Hospital University of Copenhagen Hellerup Denmark; ^3^ Department of Dermatology and Allergy Allergy Clinic Herlev and Gentofte Hospital University of Copenhagen Hellerup Denmark

**Keywords:** Allergy, aluminium, dermatitis, granuloma, LPT, SCIT, vaccine

To the Editor

Aluminium contact allergy is mainly seen in children with itching vaccination granulomas following immunization with aluminium‐adsorbed vaccines, but may also occur in adults following allergen‐specific subcutaneous immunotherapy, SCIT, as these vaccines too are aluminium‐adsorbed.^1^ Traditional method of determining sensitization to aluminium is patch testing, an in vivo skin test considered the gold standard for detecting contact allergy.^2^ Still, it has the disadvantage of only detecting the allergic response in the skin, and not systemic reactions. In adults there is a great risk of false‐negative patch test results even though new recommendations on a higher aluminium concentration, rising from 2% to 10% aluminium chloride hexahydrate (AlCl_3_H_2_O_6_), has been implemented.^3^

An alternative to patch testing is the blood in vitro lymphocyte proliferation test (LPT), also known as the lymphocyte transformation test (LTT), which we investigated using a well‐established LPT protocol. This has previously been shown to detect and characterize metal‐specific cells and was used to detect circulating aluminium‐specific proliferation. The LPT test is based on a single blood sample and has mostly been used to detect drug hypersensitivity. Still, its role in detecting metal allergy is expanding,^4^ with recent studies suggesting using the test as a supplement to the patch test when only a few allergens are to be investigated.^5^ Our study aimed to determine the diagnostic performance of LPT in adults with SCIT‐induced vaccination granulomas, and to evaluate the association between LPT and patch test reactions.

We included six participants with SCIT‐induced granulomas and 10 healthy controls. Inclusion of patients was limited by the small number of SCIT‐recipients referred to our department.

Characteristics of the participants with SCIT‐induced granulomas are shown in Table 1. All granulomas were long‐lasting, and except for one participant with 2 granulomas, all had 3 or more palpable and itching granulomas, mainly on the upper arms but also on the flanks. The control group were matched for age and sex, and did not undergo patch testing, but had no history of post‐vaccination granulomas, contact dermatitis, contact allergy, or other skin diseases, had never received SCIT and not been vaccinated with an aluminium‐adsorbed vaccine within the last year.

**TABLE 1:** Characteristics of the participants with SCIT‐induced granulomas.

ID no	Sex	Age	Granuloma			Patch test results	
			Number of palpable granulomas	Duration at LPT	VAS itch^a^	AlCl10% pet	Disc
1	F	57	>3	>10 years	4	‐^b^	‐
2	F	36	2	>10 years	4	++^b^	‐
3	M	34	>3	>10 years	2	++	‐
4	F	32	>3	>10 years	6	‐	‐
5	F	26	>3	4 years	3	‐	‐
6	M	37	>3	6 years	2	‐	‐

^a^ Itch scored with a visual analogue scale (VAS) from 0 to 10, with 10 being the worst possible itch, scored on the day of blood sampling.

^b^ Patch tested with AlCl2% pet before recommendations changed.

Participants with granulomas were all patch tested with AlCl_3_H_2_O_6_ 2% (before 2021) or 10% mixed in petrolatum and applied under Finn chambers, an aluminium Finn chamber and an empty plastic chamber as control (8 mm; Smartpractice, Phoenix, AZ, USA), all applied on the upper back and secured with Scanpor^®^ tape (Norgesplaster, Vennesla, Norway). Application time was 2 days, and patch test reactions were scored on day 2, 3–4 and 7.

Two participants with granulomas had a positive patch test reaction to aluminium AlCl_3_H_2_O_6_ either 2% or 10% pet, and none had a positive reaction to the metallic aluminium disc.

Following blood sampling, peripheral blood mononuclear cells (PBMC) were purified by gradient centrifugation from the blood of participants with SCIT‐induced granulomas and healthy subjects. After PBMC purification, the cells was stained with carboxyfluorescein diacetate succinimidyl ester (CFSE) as described before.^5^ Specific proliferation of PBMCs was seen after stimulation with the positive control antigen, tetanus, in participants with SCIT‐induced granulomas and healthy subjects (Figure 1A,B), indicating responsive PBMCs. As recommended when setting up an LTT assay,^6^ the CFSE‐labeled PBMCs were stimulated with the highest three non‐toxic concentrations (1.95, 7.8 and 31.3 μg/mL) of AlCl_3_H_2_O_6_ (Sigma, Missouri, USA) for 7 days. On day seven of culture, the PBMCs were stained with subtype and tissue‐associated marker antibodies and analysed by flow cytometry. Further description of the method is available in Supplementary Appendix.

**FIGURE 1:**
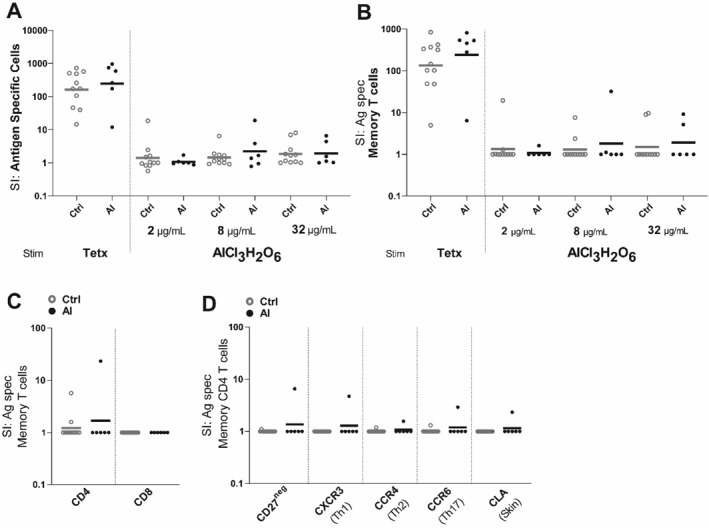
Few or no circulating aluminium‐reactive T cells in healthy subjects and participants with SCIT‐induced granulomas. PBMCs of healthy subjects (*n* = 10) and participants with SCIT‐induced granulomas (*n* = 6) were stimulated with none, tetanus toxoid, and 2, 8 and 32 μg/mL aluminium chloride for 7 days. (A–B) SI of the bulk (A) and memory T (B) antigen specific cells. Grey and black circles indicate healthy subjects and adults with granulomas, respectively. (C) SI of memory CD4 and CD8 T cells following stimulation with 8 μg/mL aluminium chloride. (D) SI of memory CD4 T cells expressing various subtypes and tissue‐homing associated markers. Ag spec, antigen‐specific; AL, participants with SCIT‐induced granulomas; Ctrl, healthy control; SI, stimulation index; Stim, stimulation.

In stimulation with three non‐toxic concentrations of aluminium, no differences were found comparing the stimulation index (SI) of proliferating (CFSE^low^) cells and memory (CD45RO+) T cells in healthy subjects and participants with SCIT‐induced granulomas (Figure 1A,B). Aluminium‐induced proliferation was only detected in the T helper (Th) cells in two healthy subjects and one participant with SCIT‐induced granulomas (Figure 1C). Only one (participant 6) of the six participants with SCIT‐induced granulomas had a specific proliferation of Th cells expressing the skin signature marker cutaneous lymphocyte‐associated antigen (CLA) (Figure 1D), although patch testing was negative.

The findings in this preliminary study indicate that there might be few or no circulating aluminium‐reactive cells in adults, despite continuing itch from the granulomas. Both the patch test and the LPT test may have low sensitivity for detecting aluminium contact allergy in adults.^4^ Other possible explanations for the negative test results are that the T cell‐dependent inflammation in the granulomas is not caused by aluminium, as granulomas have various histopathological findings,^7^ or perhaps the aluminium allergy has diminished due to the time interval between induction/elicitation and testing as reported in other studies.^8^ Moreover, the lack of specific proliferation in the LTT system could also be due to the formulation of aluminium or lagging formation of aluminium‐protein hapten complexes in vitro.

Since oral intake of aluminium may generate a systemic response with cutaneous eruptions in children with vaccination granulomas,^9^ a study on the lymphocyte reactivity characterizing the possibility of systemic reactions in these children would be of great interest.

## AUTHOR CONTRIBUTIONS

**Stine Skovbo Hoffmann**: Data curation; investigation; writing – original draft; writing – review & editing. **Jesper Elberling**: Conceptualization; supervision; writing – review & editing. **Jeanne Duus Johansen**: Conceptualization; methodology; supervision; writing – review & editing. **Lars Heede Blom**: Conceptualization; formal analysis; software; writing – review & editing.

## CONFLICT OF INTEREST STATEMENT

None.

## Supporting information

Supporting Information S1

## Data Availability

The data that support the findings of this study are available on request from the corresponding author. The data are not publicly available due to privacy or ethical restrictions.
