# Supplementary material for: Lack of detection of aluminium‐reactive T‐lymphocytes in patients with SCIT‐induced granulomas
Source: Clin Transl Allergy. 2024 Jul 2;14(7):e12378. doi: 10.1002/clt2.12378 (PMC11217595; doi:10.1002/clt2.12378)
Supplement: Supplementary file 1 — Supporting Information S1 [file CLT2-14-e12378-s001.docx]

**Supplemental Information**

LPT test

PBMCs were purified from 18 mL blood (EDTA tubes, Greiner bio-one, AT) as previously described.^1^ The erythrocytes were lysed with ammonium chloride potassium (AKC) Lysing Buffer (RH Apotek, Herlev, DK) for 2 min at room temperature (RT) in the dark. The PBMCs were stained with CFSE, as previously described.^2,3^ In short, the PBMCs were reconstituted in 990 µL PBS with 5% FCS (Invitrogen, Carlsbad, CA) and stained with 110 µL CFSE solution (Invitrogen) in a final concentration of 5 µM for 5 min at RT in the dark. The PBMCs were cultured in RPMI 1640 supplemented with l-glutamine (1 mm), *β*-mercaptoethanol (50 mm) (all from Sigma, Missouri, USA) and 10% heat-inactivated sterile filtered human AB serum (Invitrogen). The PBMCs were stimulated with; medium control, tetanus toxoid (10 µg/mL, AJ Vaccines, Copenhagen, DK), phytohemagglutinin (10 µg/mL) and AlCl_3_H_2_O_6_ (1.95, 7.8 and 31.3 µg/mL) (both from Sigma) for seven days. The PBMCs were cultured at a density of 2*10^6^ cells per mL in 1-2.5 mL. 1x of the starting volume of RPMI culture medium with 10% AB serum was added to the PHA cultures at day 4-5 of culture. Toxicity testing of AlCl_3_H_2_O_6_ drugs was conducted, with the inspiration of Pichler and Tilch,^4^ prior to LPT analysis by stimulating PBMCs of two healthy donors with 10 µg/mL PHA for seven days together with a gradient of AlCl_3_H_2_O_6_ (1000-0.06 µg/mL) (sup Figure 1). Recognizing that calculations of SI of less frequent cell populations increased the variation, a 0.01% cut-off was introduced. The cut-off value of 0.01% is calculated off the non-dead single cell population and is the lowest value used for SI calculations.

Flow cytometry

After seven days of culture, cells were stained with a viability dye (Fixable Viability Dye eFluor® 780, eBioscience, San Diego, CA) for 20 min at 4°C. Next, the cells were stained with PE-Cy7 CCR4 (1G1), APC CD161 (DX12), BV421 CLA (HECA-452), BV480 CD45RO (UCHL1), BV605 CD3 (SK7), BV711 CCR6 (11A9) and BV786 CXCR3 (1C6/CXCR3) (all from BD Biosciences, San Jose, CA) and PE CD8 (SK1, BioLegend, CA, USA) for 20 min at RT. Following, the cells were fixed in IC Fixation Buffer (Invitrogen) for 20 min at RT. The cells were analysed by an LSRfortessa (BD), and data were analysed with FlowJo software 10.8.1 (BD Biosciences).

References

1. Blom LH, Martel BC, Larsen LF, et al. The immunoglobulin superfamily member CD200R identifies cells involved in type 2 immune responses. *Allergy Eur J Allergy Clin Immunol*. Published online 2017. doi:10.1111/all.13129

2. Quah BJC, Warren HS, Parish CR. Monitoring lymphocyte proliferation in vitro and in vivo with the intracellular fluorescent dye carboxyfluorescein diacetate succinimidyl ester. *Nat Protoc 2007 29*. 2007;2(9):2049-2056. doi:10.1038/nprot.2007.296

3. Witten M, Malling HJ, Blom L, Poulsen BC, Poulsen LK. Is intralymphatic immunotherapy ready for clinical use in patients with grass pollen allergy? *J Allergy Clin Immunol*. 2013;132(5):1248-1252.e5. doi:10.1016/j.jaci.2013.07.033

4. Pichler WJ, Tilch J. The lymphocyte transformation test in the diagnosis of drug hypersensitivity. *Allergy Eur J Allergy Clin Immunol*. 2004;59(8):809-820. doi:10.1111/j.1398-9995.2004.00547.x
